# Research hotspots and trends of amblyopia treatment from 2015 to 2025: a bibliometric analysis

**DOI:** 10.3389/fnins.2025.1720376

**Published:** 2026-01-08

**Authors:** Chenyan Zhou, Xiaoru Li, Xiaoliang Luo, Shuning Song, Lvchun Wang, Junpeng Huang, Xiaobing Zhang, Die Liu, Yong Chai, Jiawei Zhou

**Affiliations:** 1 State Key Laboratory of Eye Health, Eye Hospital, Wenzhou Medical University, Wenzhou, China; 2 China Mobile Virtual Reality Innovation Center, Nanchang, China; 3 Department of Ophthalmology, Jiangxi Provincial Children’s Hospital, The Affiliated Children’s Hospital of Nanchang Medical College, Nanchang, China

**Keywords:** amblyopia, bibliometric analysis, CiteSpace, therapy, treatment

## Abstract

**Background:**

This study aims to provide a comprehensive view of the current research status, hotspots, and emerging trends of amblyopia treatment through bibliometric analysis of published literature.

**Methods:**

We retrieved publications from the Web of Science Core Collection (WoSCC) and PubMed databases from 2015 to 2025. CiteSpace was used to analyze and visualize countries, institutions, journals, keywords, and references.

**Results:**

The analysis included 514 publications that show a steady increasing trend. The United States and China are the most productive countries. McGill University has made notable contributions to this field. The most prolific journal is the Journal of AAPOS, while high-impact journal JAMA Ophthalmology has the highest citations. Keyword and reference analyses reveal that the research focus has shifted to clinical trials and various binocular treatments. The analyses of the recent citation burst and clinical trials reflect the future trend of amblyopia treatment towards personalized and technologically optimized treatment and the validation of novel neuromodulation therapies.

**Conclusion:**

This study reveals the current hotspots and future directions of amblyopia treatment research. Current research focuses on clinical trials of binocular treatment through different digital platforms in amblyopia. Our findings provide critical insights for scientific and clinical research on amblyopia treatment.

## 1 Introduction

Despite great advances in pediatric vision screening and public health, amblyopia remains the most common cause of monocular visual impairment in children worldwide. It is a neurodevelopmental disorder that arises from abnormal visual experience during the critical period of visual development (Birch, 2013; Hubel and Wiesel, 1970). With an estimated 1–5% prevalence in the general population (Attebo et al., 1998; Fu et al., 2020), this disease presents a unique clinical challenge. Along with monocular visual deficits such as decreased visual acuity and contrast sensitivity (Bradley and Freeman, 1981; Hess and Howell, 1977), it also causes binocular visual deficits including poor stereoscopic vision (Levi et al., 2015; Walraven and Janzen, 1993) and impaired binocular balance (Huang et al., 2009; Mao et al., 2020). Without timely intervention, it can lead to permanent visual deficits, severely impacting an individual’s learning, career choices, and overall quality of life (Carlton and Kaltenthaler, 2011; Davidson and Quinn, 2011).

For decades, amblyopia treatment has always been a focus of ophthalmic research and clinical practice. The traditional amblyopia treatment includes refractive correction, patching, and atropine penalization of the fellow eye (Chen and Cotter, 2016; Papageorgiou et al., 2019). These all aim to promote the use of amblyopic eye during the critical period. However, conventional therapies face numerous challenges, including low compliance and long treatment periods. Beyond these issues, their effectiveness is also limited and variable. Although these treatments are generally effective in younger children, they frequently fail to achieve a complete cure (Repka et al., 2003; Wang et al., 2021), and their efficacy is markedly reduced in older children and adults (de Zárate and Tejedor, 2007; Holmes and Levi, 2018). This reflects the critical need for more effective treatment strategies across all age groups. Driven by the development in neuroscience and technology, novel amblyopia therapies have emerged, such as perceptual learning, binocular iPad games, and non-invasive brain stimulation (El Salmawy et al., 2025; Jin et al., 2022; Kelly et al., 2016). These emerging approaches target binocular vision and extend the therapeutic window beyond early childhood. At the same time, research on the clinical application of these therapies has grown accordingly. Therefore, with the rapid growth of research in this domain, a comprehensive overview of the research status, trends, and emerging hotspots of amblyopia treatment could provide critical insight into the evolving knowledge base and future directions of the field.

In this context, bibliometric analysis provides a powerful quantitative method for mapping the current state of research in the field of amblyopia treatment. Bibliometrics is a widely used tool that integrates knowledge from bibliography, statistics, and mathematics to identify key elements of a research field, such as countries, institutions, journals, keywords, and references (Ninkov et al., 2022). It helps researchers to understand the collaborative patterns, trace evolution of research themes, and figure out future trends that might not be apparent through traditional reviews.

To date, there is currently a lack of bibliometric research in the field of amblyopia treatment. Therefore, to fill this gap in the existing literature, our study employs bibliometric methods and visualization techniques to systematically outline the current state, development trends and hotspots of amblyopia treatment research over the last ten years. We aim to provide insights for researchers and improve amblyopia management for clinicians.

## 2 Methods

### 2.1 Data source and retrieval strategy

The data of bibliometric analysis were retrieved from the Web of Science Core Collection (WoSCC) database on 22 August 2025. WoSCC is globally regarded as a high-quality database. Due to its rigorous journal selection and comprehensive citation data, this database is widely used for accurate and authoritative bibliometric analysis. The search strategy employed the following query: TS = (“amblyopia” OR “anisometropic amblyopia” OR “strabismic amblyopia” OR “lazy eye”) AND TS = (“treatment*” OR “therap*” OR “approach*”) AND PY = (2015–2025), which was designed to include all relevant studies on the treatment of amblyopia. The timeframe for this analysis (2015–2025) was selected for two primary reasons. First, it aimed to capture the most recent decade of research and provide an up-to-date overview of current trends and hotspots. Second, this period is widely recognized as a critical phase marked by the rise of various novel therapies (Cruz et al., 2023; Wallace et al., 2018), especially binocular digital therapy, making it highly relevant for studying the evolution of amblyopia treatment. This initial search yielded 1413 publications. In addition, given the pivotal role of randomized controlled trials (RCTs) in evaluating amblyopia treatment efficacy and directly contributing to amblyopia clinical guidelines, we performed a separate search of RCTs on PubMed. We used the same search strategy and the dedicated “Randomized Controlled Trial” article type filter on PubMed. A total of 53 RCTs were included for further analysis of clinical trial characteristics of amblyopia treatment.

Following PRISMA guidelines, we first excluded 62 non-English publications and 179 records that were not articles or reviews. To exclude articles that were not closely related to the research topic, a manual screening process was subsequently performed independently by two researchers based on the title, abstract and full-text when necessary. This manual screening, which led to the exclusion of 658 records, was conducted based on the following criteria. The inclusion criteria encompassed: the primary research objective was the evaluation or discussion of an amblyopia therapy. The exclusion criteria comprised: (1) wrong population: studies not involving amblyopic patients; (2) non-treatment studies: studies where amblyopia treatment was not the primary focus (e.g., focusing on etiology or diagnosis); (3) irrelevant content: amblyopia treatment was only mentioned peripherally (e.g., solely in the introduction or discussion section). The inter-rater reliability between the two researchers was assessed using Cohen’s Kappa, which showed substantial agreement (κ = 0.74). A third researcher was consulted to resolve disagreements.

### 2.2 Data analysis

The full records and cited references of the screened publications were exported. Prior to the analysis and visualization, the data were first disambiguated by standardizing institutional names, checking journal name changes, and consolidating similar expressions. The valid data were saved in download-txt format and subsequently imported into two software tools: Microsoft Excel 2021 and CiteSpace (v6.4.R1). Microsoft Excel 2021 was used to draw annual publication volume and statistical graphs. CiteSpace was employed to create network maps for countries, institutions, and keywords. In these network maps, the node size represents the frequency of its occurrence. The centrality of a node quantifies the importance of its position in the network. This was measured using betweenness centrality (BC), which calculates the proportion of shortest paths in the network that pass through a given node (Brandes, 2001; Freeman, 1979). High centrality values reflect that the node plays a key role in information transfer and resource sharing in the network. A centrality value exceeding 0.1 indicates that the node acts as a crucial hub within the network. The line between nodes indicates that they appeared in the same publication (Barabasi et al., 2002; Sabe et al., 2022). Specifically, for countries and institutions, it represents a collaborative relationship via co-authorship; for keywords, it indicates a thematic association. The width and color of the lines represent the connection strength and the time of initial connection, respectively. Furthermore, CiteSpace was also used to analyze keyword clustering and citation bursts in keywords and references to identify major research themes and evolving trends. The keyword clusters were generated using a modularity-based algorithm that identifies groups of tightly connected keywords. In the cluster map, the modularity (Q score) is a metric that evaluates the extent to which a network can be divided into distinct clusters or modules (Newman, 2006). A Q score above 0.3 signifies a significant community structure. The silhouette (S score) is a metric that assesses the quality and homogeneity of the clustering results by measuring how similar an object is to its own cluster compared to other clusters (Newman, 2006). A S score greater than 0.7 indicates a strong and convincing cluster. The citation bursts analysis identifies keywords or references that experience a sharp increase in citation frequency over a specific period, which helps trace the temporal dynamics of research hotspots (Chen et al., 2014; Chen et al., 2010; Kleinberg, 2002).

## 3 Results

### 3.1 Analysis of publications

**FIGURE 1:**
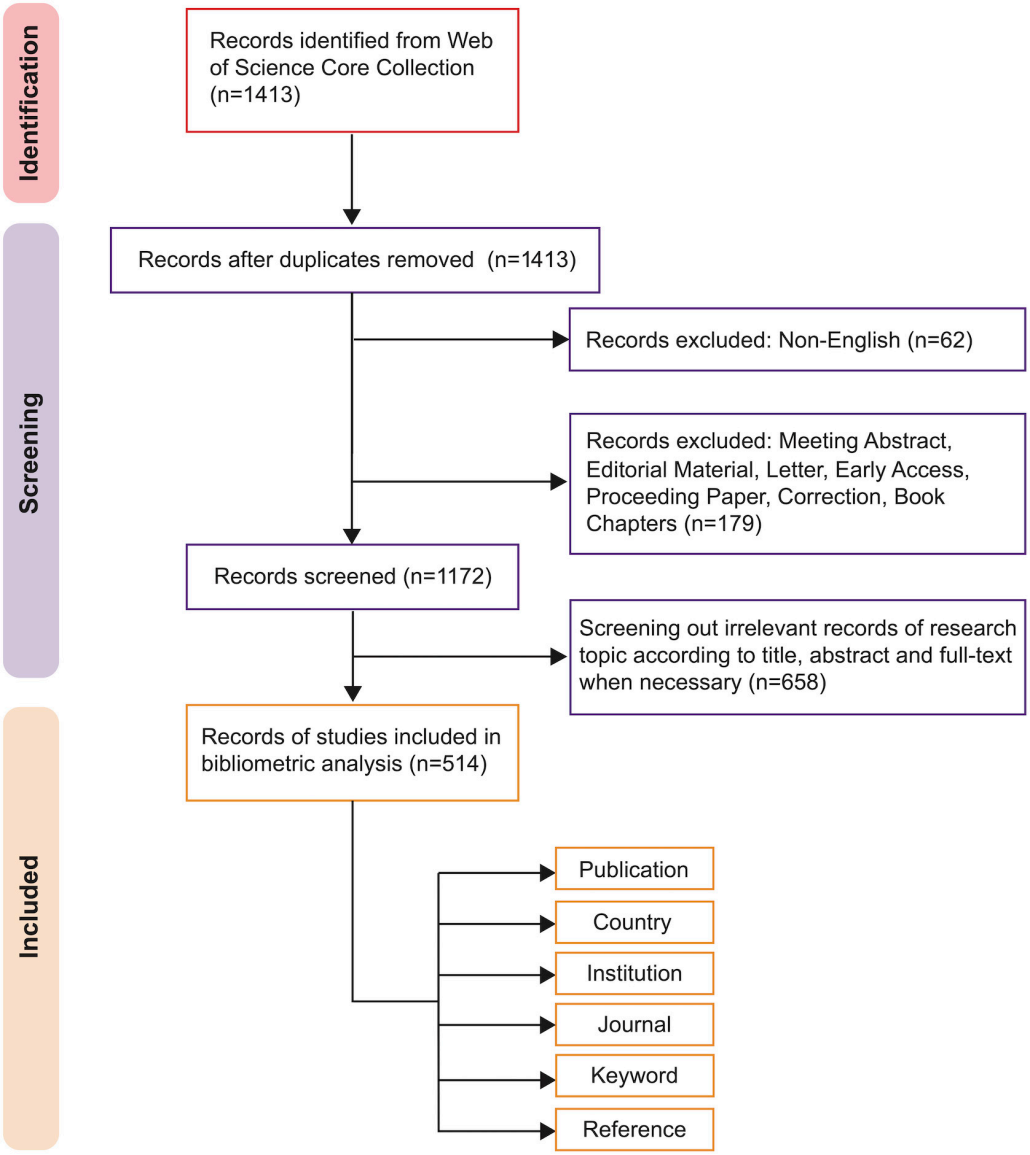
PRISMA flow chart for the literature search and screening.

From 2015 to 2025, a total of 514 publications were finally identified in WoSCC, including 422 articles and 92 reviews. The screening procedure is illustrated in Figure 1.

**FIGURE 2:**
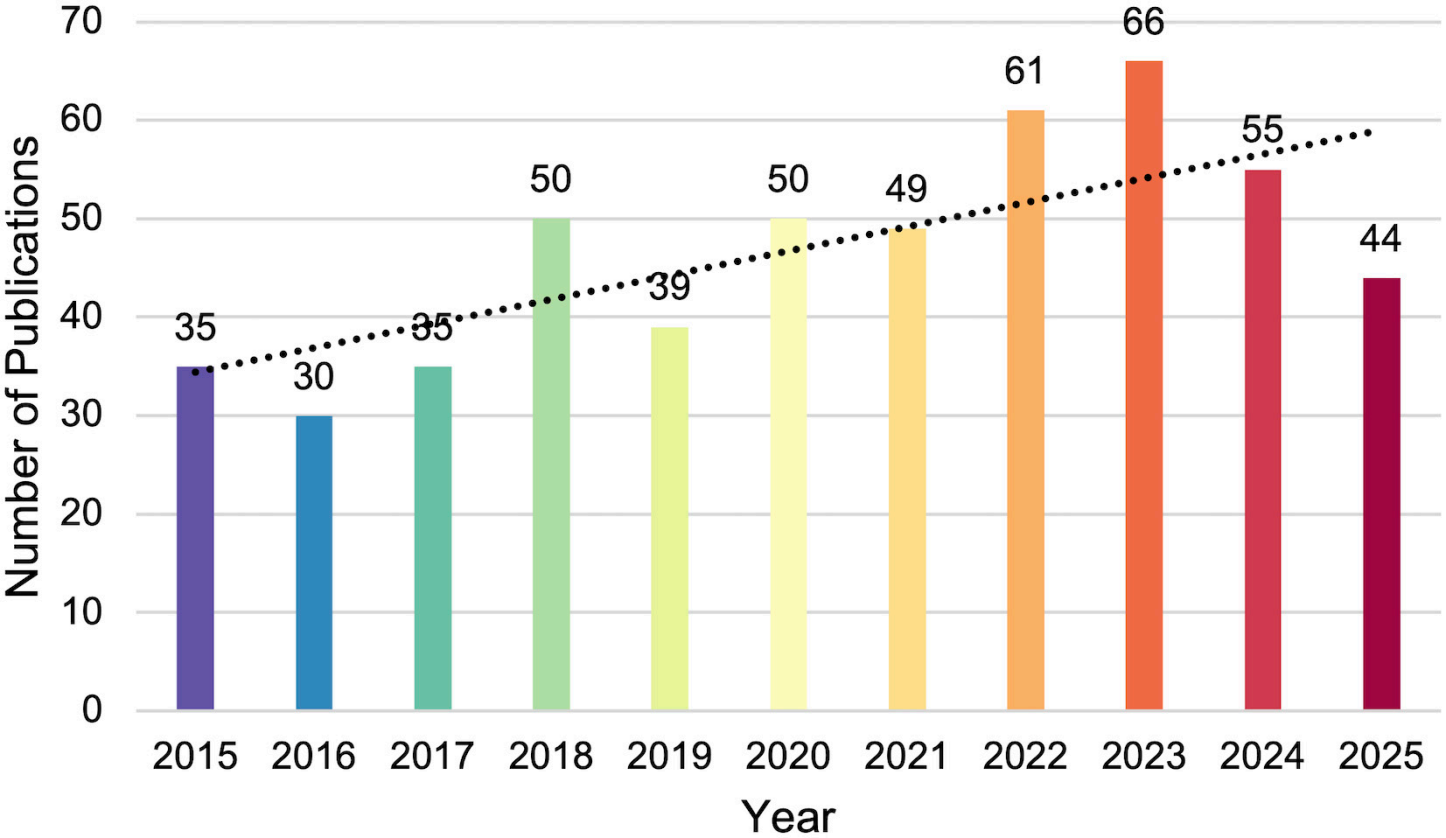
Annual publication trends in the treatment for amblyopia from 2015 to 2025. Each bar color corresponds to a specific publication year, which aligns with the color coding adopted for timeline representations in the following figures. The dotted line indicates the linear trend of publication growth over the period.

The number of annual publications can reflect the extent of academic attention to this specific research field and predict its potential trends. As shown in Figure 2, the number of publications on amblyopia treatment has steadily increased from 2015 to 2025, despite slight decreases in 2019 and 2024. The publication volume reached a peak of 66 in 2023, nearly doubling the output in 2015. The steady increase in annual publications on amblyopia therapy reflects that it remains a continuously and actively evolving field of research in the last decade.

### 3.2 Analysis of countries

**FIGURE 3:**
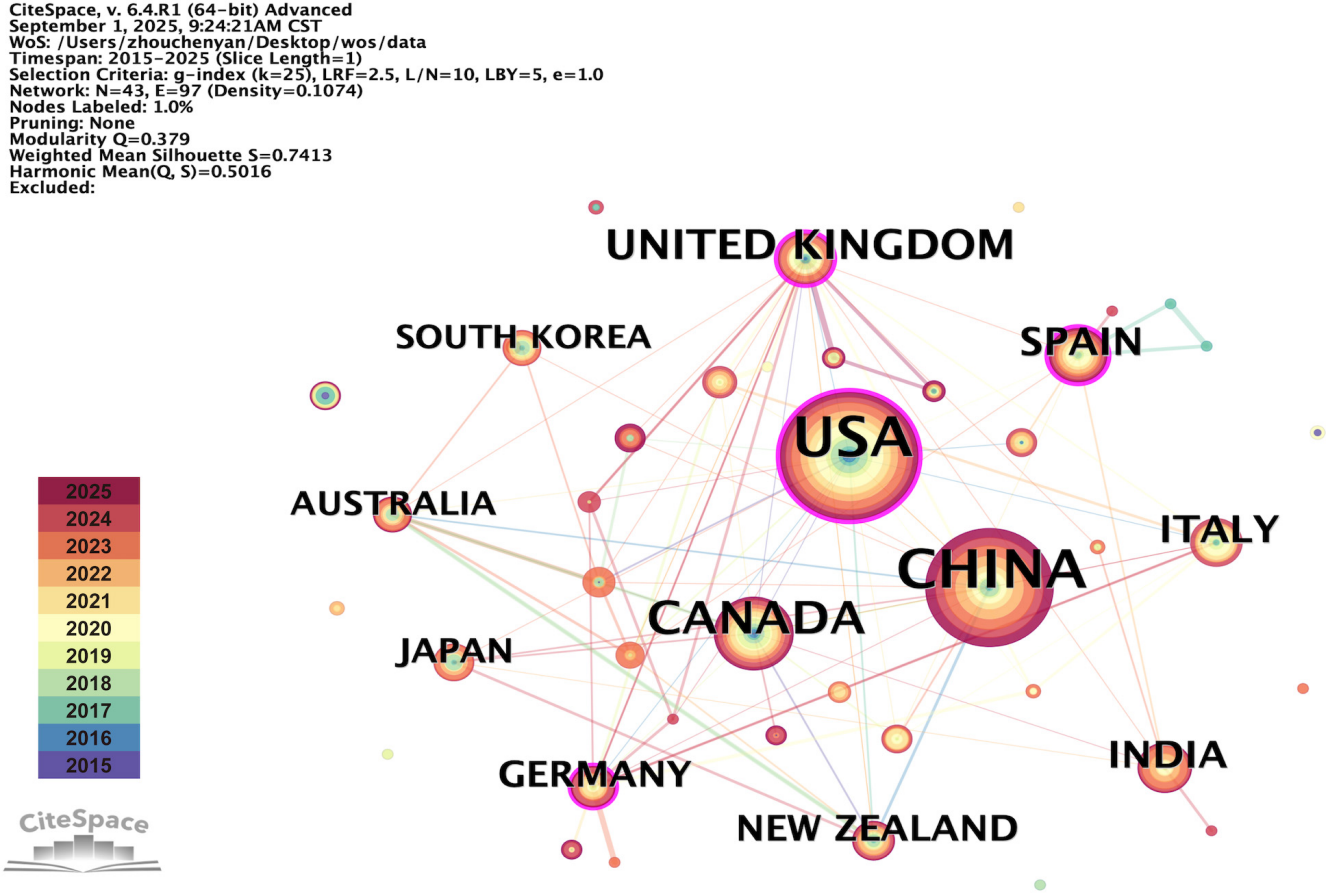
Network visualization of countries contributed to the treatment for amblyopia. Each colored node represents a country, with larger node areas indicating higher publication output. Node color corresponds to publication year (see legend), which is consistent with Figure 2. Nodes with a purple ring have a centrality value > 0.1, indicating their role as key hubs in the network. Each line connecting two nodes indicates a collaborative relationship, which was defined as both countries having authors listed on the same publication. The color and width of the lines represent the year of initial collaboration and its strength.

From 2015 to 2025, a total of 43 countries contributed to the research of amblyopia treatment. Table 1 shows the top 10 productive countries, along with their corresponding publication counts and centrality. Figure 3 illustrates the cooperative networks between these countries, with nodes of centrality exceeding 0.1 highlighted by a purple ring. The top 3 countries with the highest publication counts are the United States (*n* = 165), China (*n* = 140), and Canada (*n* = 60). More than half (59.34%) of all publications are from the United States and China. However, the top 3 countries with the highest centrality are the United Kingdom (BC = 0.40), Spain (BC = 0.22), and the United States (BC = 0.21). This indicates that although the United States and China dominate amblyopia treatment research with the largest publication volume, the United Kingdom and Spain are important hubs in the global academic network considering their stronger connective influence.

**TABLE 1:** Top 10 productive countries in the treatment for amblyopia.

Rank	Country	Publications	Percentage	Centrality
1	USA	165	32.10%	0.21
2	China	140	27.24%	0.05
3	Canada	60	11.67%	0.09
4	UK	39	7.59%	0.40
5	Spain	30	5.84%	0.22
6	India	27	5.25%	0.09
7	Italy	27	5.25%	0.01
8	Germany	21	4.09%	0.15
9	New Zealand	21	4.09%	0.01
10	Japan	18	3.50%	0.01

### 3.3 Analysis of institutions

**FIGURE 4:**
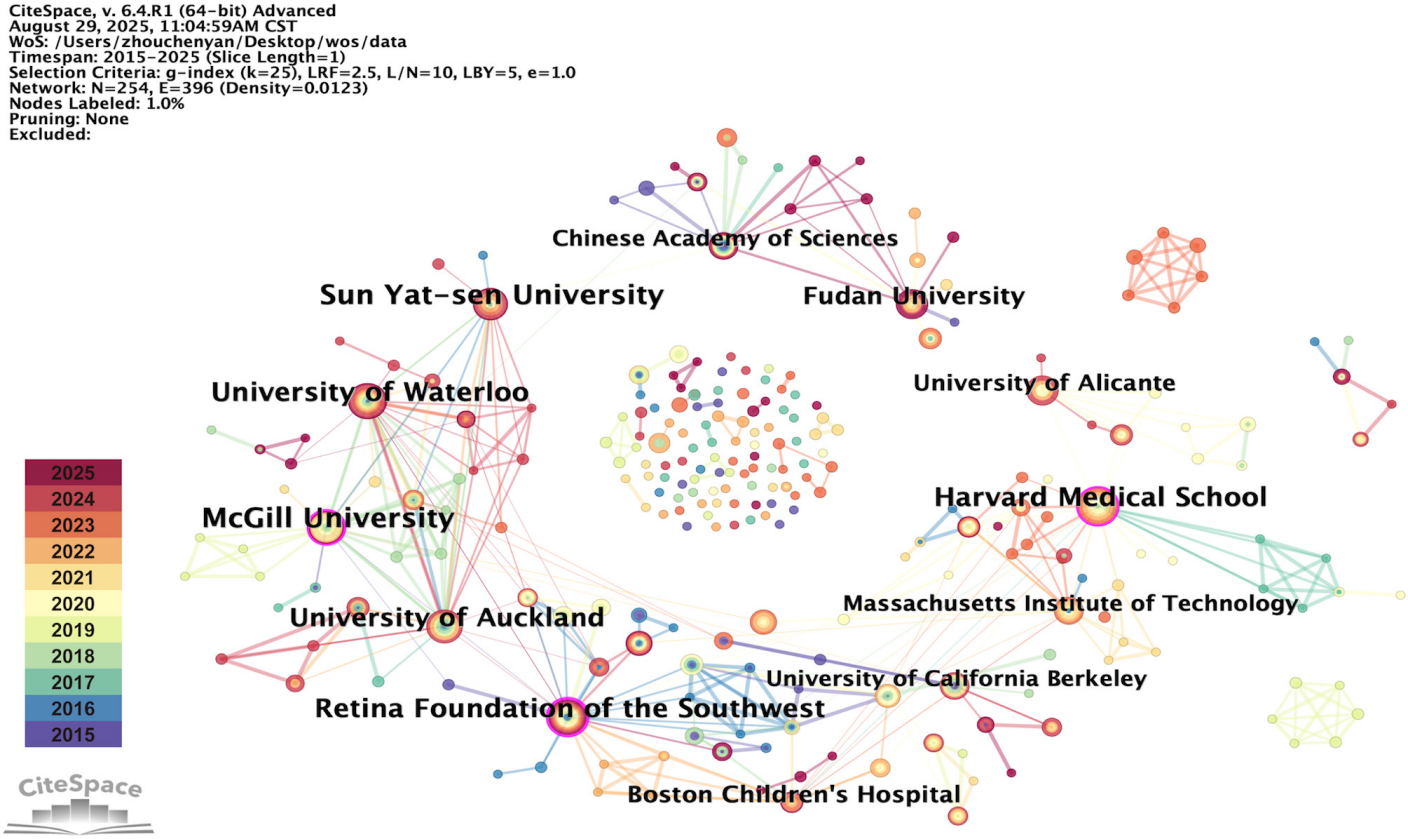
Network visualization of institutions contributed to the treatment for amblyopia. Each colored node represents an institution, with larger node areas indicating higher publication output. Node color corresponds to publication year (see legend), which is consistent with Figure 2. Nodes with a purple ring have a centrality value > 0.1, indicating their role as key hubs in the network. The small nodes at the center represents institutions with both low publication output and low network centrality. Each line connecting two nodes indicates a collaborative relationship, which was defined as both institutions having authors listed on the same publication. The color and width of the lines represent the time of initial collaboration and its strength.

A total of 254 institutions were included in this study. Table 2 lists the top 10 productive institutions, along with their corresponding publication year, publication counts, and centrality. Figure 4 visualizes the collaborative relationships among these institutions. The node with a centrality exceeding 0.1 is marked by a purple ring. McGill University (*n* = 22) and Sun Yat-sen University (*n* = 22) have the most publications, followed by Harvard Medical School (*n* = 20) and University of Waterloo (*n* = 20). The centrality of Retina Foundation of the Southwest (BC = 0.21), McGill University (BC = 0.17), and Harvard Medical School (BC = 0.13) all exceed 0.1, indicating that these institutions play significant roles in the collaboration (Figure 4). As shown in the figure, institutional collaboration is mainly concentrated in North American, forming a complex cooperative network. Among these institutions, McGill University not only has substantial publication output but also shows high network centrality, demonstrating its important contribution to both research production and knowledge exchange. Additionally, the Retina Foundation of the Southwest has the highest centrality, reflecting its crucial role in transferring information across the network. Furthermore, Chinese institutions exhibit diverse and continuously evolving collaboration patterns. Some institutions, represented by Sun Yat-sen University, are deeply incorporated into the global collaboration network, while others, represented by Fudan University and the Chinese Academy of Sciences, are relatively isolated in the global network but have increasingly strong domestic collaboration.

**TABLE 2:** Top 10 productive institutions in the treatment for amblyopia.

Rank	Institution	Publications	Percentage	Centrality
1	McGill University	22	4.28%	0.17
2	Sun Yat-sen University	22	4.28%	0.07
3	Harvard Medical School	20	3.89%	0.13
4	University of Waterloo	20	3.89%	0.06
5	Retina Foundation of the Southwest	19	3.70%	0.21
6	University of Auckland	17	3.31%	0.06
7	Fudan University	15	2.92%	0.04
8	Boston Children’s Hospital	13	2.53%	0.05
9	University of Alicante	12	2.33%	0.04
10	Massachusetts Institute of Technology	11	2.14%	0.06

### 3.4 Analysis of journals

Tables 3, 4 list the top 10 journals by the number of publications and citations, respectively. There is a clear distinction between the highly productive and influential journals. Most research output is from specialized journals such as the Journal of AAPOS (*n* = 28), which focuses on pediatric ophthalmology and strabismus. This indicates its role as a central platform for researchers in this field to share their findings. However, in terms of citations, the top comprehensive ophthalmology journals in the Q1 quartile of JCR lead the field, including JAMA Ophthalmology (538 citations), Investigative Ophthalmology & Visual Science (420 citations), and Ophthalmology (402 citations). This suggests that while specialized journals lead in publication volume, the most influential and pioneering work that shapes research directions tend to appear in high-impact comprehensive journals. In addition, the Journal of AAPOS and Investigative Ophthalmology & Visual Science rank highly in both lists, indicating their unique positions as highly productive and influential journals in this field.

**TABLE 3:** Top 10 productive journals in the treatment for amblyopia.

Rank	Journal	Publications	Percentage	IF (2024)	JCR
1	Journal of AAPOS	28	5.45%	1.3	Q3
2	Investigative Ophthalmology & Visual Science	22	4.28%	4.7	Q1
3	BMC Ophthalmology	22	4.28%	1.7	Q3
4	Scientific Reports	21	4.09%	3.9	Q1
5	Vision Research	18	3.50%	1.4	Q3
6	Indian Journal of Ophthalmology	16	3.11%	1.8	Q3
7	Graefe’s Archive for Clinical and Experimental Ophthalmology	15	2.92%	2.3	Q2
8	International Journal of Ophthalmology	13	2.53%	1.8	Q3
9	Clinical and Experimental Optometry	13	2.53%	1.5	Q3
10	Journal of Pediatric Ophthalmology & Strabismus	13	2.53%	0.9	Q4

**TABLE 4:** Top 10 highly cited journals in the treatment for amblyopia.

Rank	Journal	Citations	IF (2024)	JCR
1	JAMA Ophthalmology	538	9.2	Q1
2	Investigative Ophthalmology & Visual Science	420	4.7	Q1
3	Ophthalmology	402	9.5	Q1
4	Journal of AAPOS	371	1.3	Q3
5	British Journal of Ophthalmology	342	3.5	Q1
6	Vision Research	338	1.4	Q3
7	American Journal of Ophthalmology	291	4.2	Q1
8	Eye	235	3.2	Q1
9	Strabismus	227	0.8	Q4
10	Optometry and Vision Science	225	1.8	Q3

### 3.5 Analysis of keywords

#### 3.5.1 Analysis of keywords co-occurrence and clusters

**FIGURE 5:**
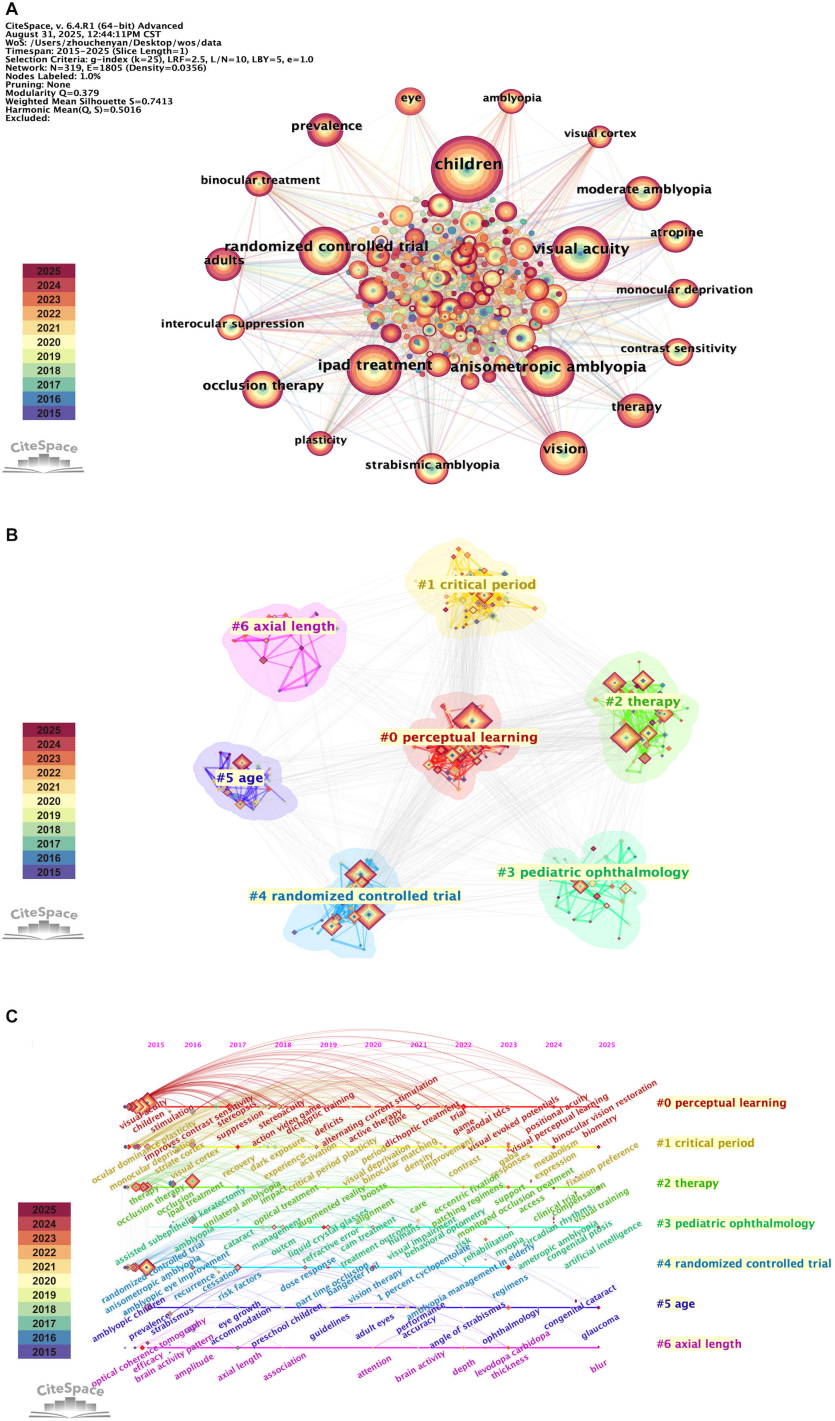
Visualization map of keywords contributed to the treatment for amblyopia. Each colored node represents a keyword, with larger node areas indicating higher frequency of occurrence. Node color corresponds to publication year (see legend), which is consistent with Figure 2. **(A)** Network visualization of keywords. Each line connecting two nodes indicates an association, which was defined as the co-occurrence of two keywords within the same publication. The color and width of the lines represent the time of initial association and its strength. **(B)** Cluster analysis of keywords. Keywords are algorithmically grouped into colored clusters that represent distinct research themes. **(C)** Timeline view of keywords co-occurrence. Keywords are algorithmically grouped into colored clusters, each labeled on the right side of the map. Each node positioned on the horizontal timeline according to the year of keyword’s first appearance. The lines between nodes indicate co-occurrence relationships, with thicker lines denoting stronger connections.

Figure 5A shows the keyword co-occurrence network. The size of the circle is proportional to the frequency of the keyword. The top 3 most frequent keywords are “children,” “visual acuity,” and “anisometropic amblyopia,” followed by “iPad treatment,” “randomized controlled trial,” “vision,” and “occlusion therapy.” This indicates that greater research attention has been focused on these topics in the last decade. Keyword cluster network is illustrated in Figure 5B. The modularity value (Q = 0.379 > 0.3) and mean silhouette value (S = 0.7413 > 0.7) of the clustering verify that the results are reliable and credible. Figure 5C further shows the timeline distribution of the keyword cluster, demonstrating the evolution of research trends in this field. Cluster #0 (perceptual learning) and Cluster #1 (critical period) focus on the visual cortex plasticity in amblyopia. The timeline view reveals that both clusters emerged early and remain active throughout the period. This sustained attention confirms that critical period is an important factor in amblyopia treatment efficacy, while perceptual learning is a promising approach to extend treatment possibilities beyond conventional limits of age. Cluster #2 (therapy) and Cluster #4 (randomized controlled trial) clearly show the evolution of amblyopia treatment and its clinical validation. The “therapy” cluster shows increasingly advanced interventions, shifting from conventional method “occlusion therapy” gradually toward “iPad treatment,” “augmented reality,” and “vision therapy.” Concurrently, research focus has expanded to treatment regimen optimization and adherence management, which is also manifested in “randomized controlled trial” cluster. Finally, Cluster #3 (pediatric ophthalmology), Cluster #5 (age), and Cluster #6 (axial length) exhibit the clinical context related to amblyopia treatment. The “pediatric ophthalmology” cluster shows the main clinical background of amblyopia which is frequently associated with diseases like cataract and strabismus. The “age” and “axial length” clusters demonstrate the importance of amblyopia screening, treatment initiation, and treatment safety.

#### 3.5.2 Analysis of keywords citation burst

**FIGURE 6:**
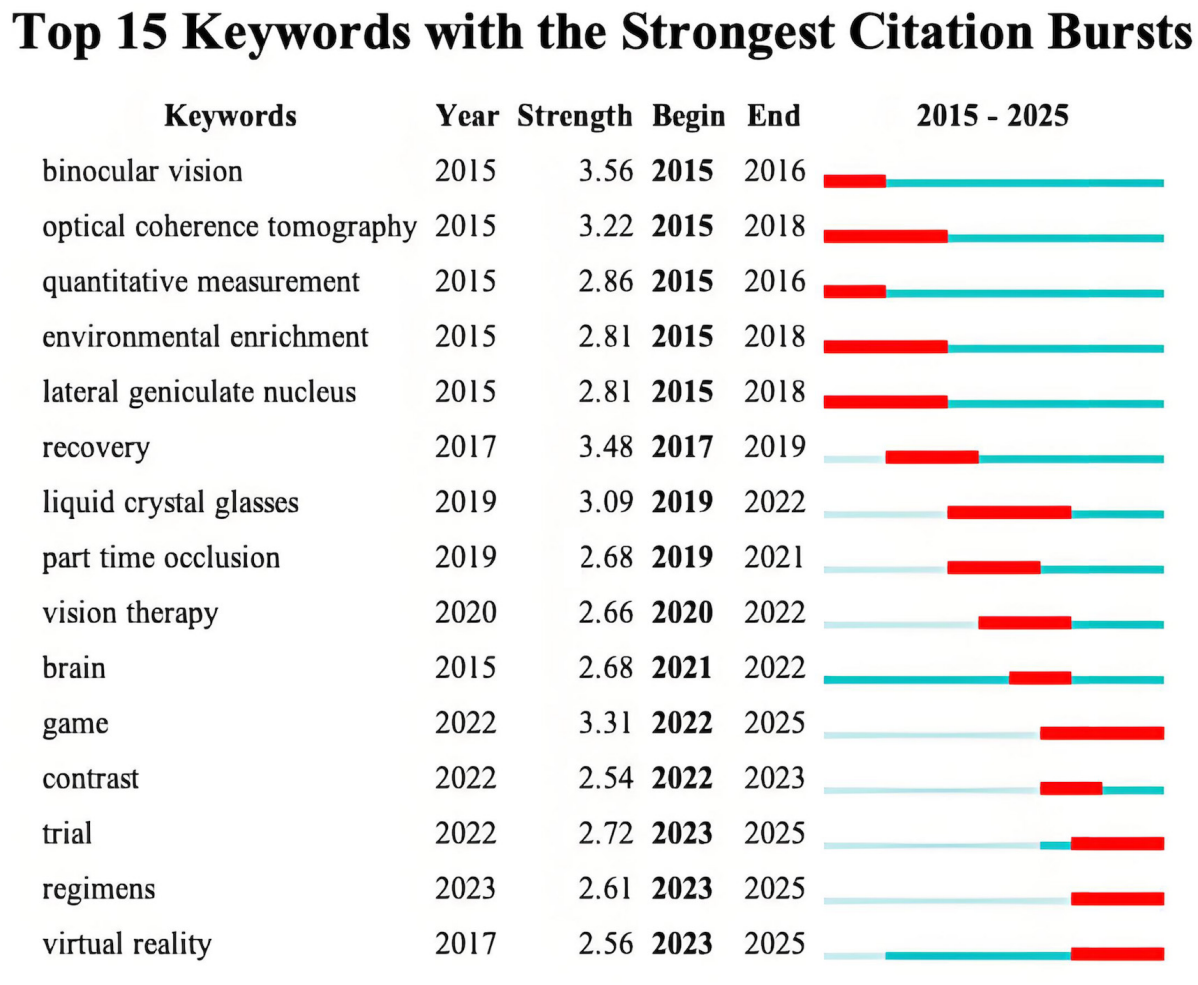
Top 15 keywords with the strongest citation bursts in the treatment for amblyopia. Year indicates the first appearance of the keyword. Begin and End indicate the duration of the keyword burst. Strength refers to the intensity of the burst, reflecting its statistical influence. The timeline for each keyword is shown horizontally. The period before the keyword’s first appearance is represented by the light blue bar. The dark blue bar represents the period from the keyword’s first appearance to the present. Within this period, the years of its citation burst are represented by the red bar. A dark blue bar starting after a red bar indicates the keyword appeared simultaneously with its burst.

Analysis of keywords citation burst reveals the research hotspots of amblyopia treatment over time. Figure 6 presents the top 15 keywords with the strongest citation bursts. Notably, the keyword “binocular vision” has the highest burst strength, verifying its importance in both understanding and treating amblyopia. Between 2015 and 2018, keywords such as “optical coherence tomography” and “lateral geniculate nucleus” indicate the research emphasis on advanced imaging techniques and neural mechanisms underlying amblyopia. From 2019 to 2022, research focus shifted toward the development of new treatment methods. The emergence of keywords including “liquid crystal glasses,” “part time occlusion,” “vision therapy,” and “game,” reflects growing interest in optimizing monocular treatments, advancing binocular therapy, and developing digital therapeutic interventions. For instance, “liquid crystal glasses” refers to a technology for intermittent occlusion, where the lens before the non-amblyopic eye electronically switches between transparent and opaque states to improve compliance with traditional patching (Spierer et al., 2010). Recently (2022–2025), keywords such as “trial,” “regimens,” and “virtual reality,” signal the trend toward evidence-based treatment optimization. These terms reveal the efforts to improve treatment strategies and validate new technologies in the treatment of amblyopia.

### 3.6 Analysis of references

#### 3.6.1 Analysis of highly cited references

Table 5 presents the top 10 highly cited references in this field. It is clearly demonstrated that the highly cited references are dominated by randomized clinical trials evaluating binocular digital therapies, especially binocular iPad games. Most of them were directly compared to traditional patching and published in high-impact journals. These findings indicate the great importance of rigorous and evidence-based testing of digital treatment in the field currently. Furthermore, the focus of these trials on older children and adults suggests a research effort on expanding treatment options beyond the conventional critical period.

**TABLE 5:** Top 10 highly cited references in the treatment for amblyopia.

Rank	Cited references	Citations	First author	Journal	Year
1	A Randomized Trial of a binocular iPad Game Versus Part-Time Patching in Children Aged 13 to 16 Years with Amblyopia	51	Manh, Vivian M	American Journal of Ophthalmology	2018
2	A Randomized Trial of Binocular Dig Rush Game Treatment for Amblyopia in Children Aged 7 to 12 Years	50	Pediatric Eye Disease Investigator Group	Ophthalmology	2019
3	Binocular iPad Game vs. Patching for Treatment of Amblyopia in Children: A Randomized Clinical Trial	50	Kelly, Krista R	JAMA Ophthalmology	2016
4	Effect of a Binocular iPad Game vs. Part-time Patching in Children Aged 5 to 12 Years with Amblyopia: A Randomized Clinical Trial	45	Holmes, Jonathan M	JAMA Ophthalmology	2016
5	Effectiveness of a Binocular Video Game vs. Placebo Video Game for Improving Visual Functions in Older Children, Teenagers, and Adults with Amblyopia: A Randomized Clinical Trial	43	Gao, Tina Y	JAMA Ophthalmology	2018
6	Binocular iPad treatment for amblyopia in preschool children	42	Birch, Eileen E	Journal of AAPOS	2015
7	Randomized Controlled Trial of a Dichoptic Digital Therapeutic for Amblyopia	40	Xiao, Scott	Ophthalmology	2022
8	Dichoptic Training Enables the Adult Amblyopic Brain to Learn	39	Li, Jinrong	Current Biology	2013
9	Rethinking amblyopia 2020	37	Levi, Dennis M	Vision Research	2020
10	Global prevalence of amblyopia and disease burden projections through 2040: a systematic review and meta-analysis	35	Fu, Zhujun	British Journal of Ophthalmology	2020

#### 3.6.2 Analysis of references citation burst

**FIGURE 7:**
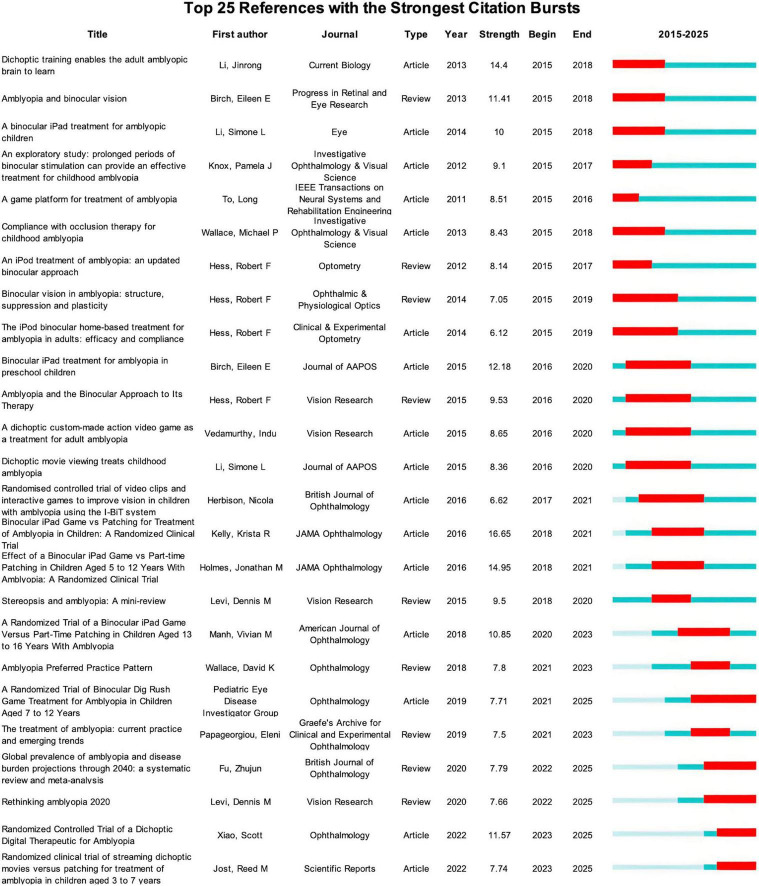
Top 25 references with the strongest citation bursts in the treatment for amblyopia. Year indicates the publication year. Begin and End indicate the duration of the reference burst. Strength refers to the intensity of the reference burst, reflecting its statistical influence. The timeline for each reference is shown horizontally. The light blue bar represents the period prior to the reference’s publication. The dark blue bar represents the period from the reference’s publication to the present. Within this period, the years of its citation burst are represented by the red bar. A dark blue bar starting after a red bar indicates the reference appeared simultaneously with its burst.

Analysis of reference burst strength reveals the evolution of knowledge framework in this field. Figure 7 presents the top 25 references with the strongest citation bursts. From 2015 to 2018, the references with the strongest citation bursts were mainly the research exploring neural mechanisms and plasticity of amblyopia. For example, the paper entitled “Amblyopia and binocular vision” (Birch, 2013) and “Binocular vision in amblyopia: structure, suppression and plasticity” (Hess et al., 2014) highlight the important role of binocular vision damage and its underlying mechanism in amblyopia. These highly cited publications laid an essential theoretical foundation for subsequent innovations in treatment methods. Subsequently, the research emphasis began shifting toward various binocular treatments, including binocular iPad games (Birch et al., 2015) and dichoptic movie viewing (Li et al., 2015). These studies provided initial evidence for the efficacy of these novel interventions. Recently, reference bursts become notably concentrated on randomized controlled trials, especially dichoptic digital therapy (Jost et al., 2022; Pediatric Eye Disease Investigator Group et al., 2019). This indicates the field has entered an evidence-based phase of clinical validation of novel treatment.

### 3.7 Analysis of clinical trials

The bibliometric analysis above highlighted the foundational role of RCTs in this field, as evidenced by the high frequency of the keyword “randomized controlled trial” and the dominance of RCTs among the most highly-cited publications. To move beyond quantifying their influence and to qualitatively explore the specific characteristics of this clinical evidence base, we conducted a detailed analysis of 53 RCTs retrieved from PubMed. This supplementary analysis aimed to delineate the evolution of amblyopia therapies, patient populations, and outcome measures based on clinical evidence.

**FIGURE 8:**
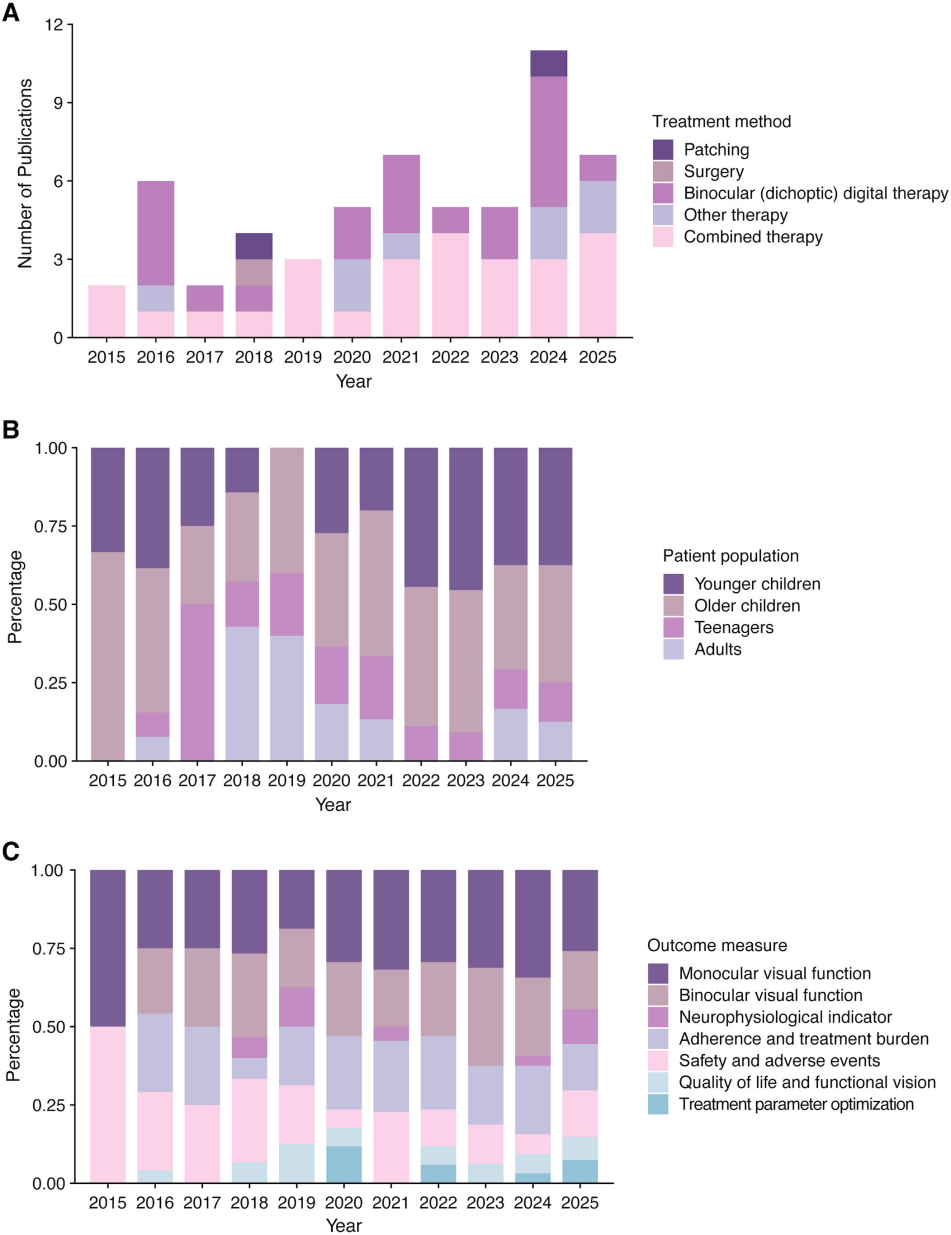
Trends in amblyopia RCTs retrieved from PubMed from 2015 to 2025. The x-axis in all panels represents the publication year. Each colored segment represents a distinct category of treatment method, patient population, or outcome measure, as detailed in the legend adjacent to each panel. **(A)** Evolution of treatment methods. The y-axis represents the annual number of RCTs investigating each primary therapy. **(B)** Shifts in patient populations. The y-axis represents the annual proportion of RCTs including each age group. **(C)** Diversification of outcome measures. The y-axis represents the annual proportion of RCTs that assessed each outcome domain.

We first categorized the primary treatment method of RCTs according to Amblyopia Preferred Practice Pattern^®^ (PPP) guidelines (Figure 8A; Cruz et al., 2023). The analysis reveals that clinical research focus remains strongly on dichoptic digital therapy and combined treatment (particularly combined with patching) in the last decade. Much of this effort has been directed toward establishing the efficacy of dichoptic therapy against traditional patching, or toward identifying optimal combination regimens where patching effectiveness is augmented. Recently, a growing body of work has emerged to clinically validate novel therapeutic approaches, including transcranial direct current stimulation (tDCS) (El Salmawy et al., 2025), syntonic phototherapy (Mohamed et al., 2025), and transcranial magnetic stimulation (TMS) (Lin and Cai, 2025). The exploration of neuromodulation techniques such as tDCS and TMS reflects a growing interest in directly modulating neural plasticity to extend the treatment window. Together, these trends highlight an ongoing shift toward diversification in amblyopia treatment.

Concurrently, the patient population in clinical trials broadened. We categorized the patient populations in RCTs into four groups: younger children (<7 years), older children (7 to < 12 years), teenagers (12 to < 18 years), and adults (≥18 years), and calculated the annual proportion of trials involving each group in Figure 8B. Firstly, it is revealed that trials enrolling older children, teenagers, and adults collectively constituted the majority of the clinical research compared to those enrolling younger children. This demonstrates that therapeutic development of amblyopia is no longer predominantly confined to the classical critical period. In addition, it is shown in the figure that there are two interest bursts in treatment for amblyopic adults. From 2018 to 2021, this burst was mainly associated with therapies combining patching and pharmacological treatment, such as fluoxetine (Sharif et al., 2019) and citalopram (Lagas et al., 2019). More recently, from 2024 to 2025, it is related to the exploration of novel neuromodulation techniques, such as tDCS (El Salmawy et al., 2025) and TMS (Lin and Cai, 2025). This sustained and evolving research focus indicates that treating amblyopia in older patients is now a rapidly advancing frontier in amblyopia research.

The outcome measures of all RCTs were categorized into several distinct domains: monocular visual function, binocular visual function, neurophysiological indicator, adherence and treatment burden, safety and adverse events, quality of life and functional vision, and treatment parameter optimization. The stacked bar chart in Figure 8C illustrates the annual proportion of trials that included each measure as one of outcomes. In 2015, trial endpoints were confined to monocular visual function and safety. A clear trend of diversification emerged thereafter, with a growing proportion of trials incorporating assessments of binocular visual function as well as adherence and treatment burden. Despite this expansion in the variety of endpoints, two critical domains, patient quality of life and functional vision, and treatment parameter optimization, received consistently low attention. This analysis indicates that the scope of outcome measures in amblyopia trials has broadened over the past decade, yet increased attention to some underrepresented domains remains warranted.

## 4 Discussion

### 4.1 General information

This study conducted bibliometric analysis on publications related to amblyopia treatment from 2015 to 2025. By analyzing and visualizing through CiteSpace, our study elucidates the cooperative relationships, evolving trends, and research hotspots in this field. A sustained academic effort to address the challenges of this prevalent childhood disease is reflected by the steady increase in annual publications (Figure 2).

In the analysis of global collaborations, the United States and China contribute the majority of publications (Table 1), while the United Kingdom and Spain stand out as crucial hubs in the network (Figure 3). This separation can be partly explained by the types of resources each region utilized. The high publication output from the US and China may be related with their access to large patient populations, enabling the recruitment for the large-scale clinical trials. Meanwhile, the central role of the UK and Spain may derive from their leadership in coordinating neighboring European countries in this field. Future efforts should intentionally enhance collaboration between these countries with high productivity and centrality to maximize research efficiency and accelerate the translation of findings.

In terms of institutions, McGill University shows both high publication output and network centrality (Table 2), demonstrating its sustained and profound influence on the amblyopia treatment research. Retina Foundation of the Southwest has the highest centrality among all institutions, acting as a critical connector within the collaborative network. In addition, institution analysis reveals diverse collaboration patterns among Chinese institutions. Although Sun Yat-sen University is deeply engaged in global network, other institutions maintain relatively stronger domestic collaboration (Figure 4). Therefore, China should strengthen cooperation in international institutions in the future to enhance its international influence in the field of amblyopia treatment. Beyond individual institutions, large collaborative consortia have also been instrumental and cannot be ignored. A prime example is the Pediatric Eye Disease Investigator Group (PEDIG), which is a collaborative network comprising numerous clinical institutions mostly within the United States. PEDIG’s landmark randomized trials, such as the seminal studies comparing a binocular iPad game to patching (Holmes et al., 2016) and evaluating binocular Dig Rush game in older children (Pediatric Eye Disease Investigator Group et al., 2019), are prominently featured among the most highly cited references (Table 5) and those with the strongest recent citation bursts (Figure 7). This underscores the important role of PEDIG in shaping both research landscape and clinical guidelines for amblyopia treatment.

Analysis of journals shows a distinction between those with high publication output and citations. The Journal of AAPOS dominates in article volume (Table 3), indicating its important role as a main publication platform for the research community. In contrast, despite publishing fewer articles, high-impact journals like JAMA Ophthalmology and Investigative Ophthalmology & Visual Science (Table 4) get the majority of citations. This demonstrates that the most influential studies are typically spread through these prestigious journals. These highly cited works provide crucial knowledge bases for further research and clinical developments in amblyopia treatment.

Through keyword co-occurrence analysis, we capture the core focus of amblyopia treatment research, as reflected in the three most frequent keywords “children,” “visual acuity,” and “anisometropic amblyopia.” These terms delineate the primary patient population and the gold-standard outcome measure in this field. Notably, the prominence of “anisometropic amblyopia” underscores the importance of this subtype in amblyopia treatment research. This likely reflects its status as a common form of amblyopia in clinical practice (Fu et al., 2020; Hashemi et al., 2019).

Analysis of the keyword cluster timeline (Figure 5C) and citation burst (Figure 6) reveals the hotspots of binocular digital therapy as the majority of trending keywords are directly associated with it. Reference citation analysis aligns closely with this finding. The highly-cited (Table 5) and recently burst references (Figure 7) consist largely of clinical trials evaluating binocular digital treatment, especially binocular iPad games. In addition, these publications are often published in high-impact ophthalmology journals, reflecting the widespread recognition and attention on this treatment. Together, these findings demonstrate that binocular digital therapy is reshaping both theoretical understanding and treatment strategies of amblyopia.

### 4.2 Hotspots and trends

The keyword and reference analyses reveal the evolutionary trajectories of this field. The co-occurrence and burst analyses of keywords indicate that early work focused on the mechanisms of amblyopia damage and plasticity, while recent efforts have shifted toward creating and clinically validating novel treatment methods, especially binocular digital therapies. The recent burst of keywords such as “trial,” “regimens,” and “virtual reality” and the burst of high-citation clinical trials, reflect that the field has developed into a period of evidence-based optimization and validation of these new therapies.

Early research of amblyopia treatment in the past decade focused on revealing the mechanism of amblyopic damage at the binocular level. This hotspot is reflected by the burst of keywords such as “binocular vision” and “quantitative measurement” and references “Amblyopia and binocular vision” (Birch, 2013) and “Binocular vision in amblyopia: structure, suppression and plasticity” (Hess et al., 2014) during that period. It was initially believed that amblyopes were structurally monocular and had no useful binocular vision. However, subsequent research found that intact binocular processes do exist in amblyopia, but are suppressed under binocular viewing conditions (Hess et al., 2014; Levi et al., 2015; Zhou et al., 2018). Moreover, suppression plays an important role in both monocular and binocular deficits in amblyopic patients (Li et al., 2011; Webber et al., 2020). These insights not only advanced our understanding of the mechanism of amblyopia but also provided a theoretical basis for developing new approaches. These therapies aim to not merely train the amblyopic eye, but also to directly reduce interocular suppression and train the brain to integrate signals from both eyes (Bossi et al., 2017; Hess and Thompson, 2015). For example, dichoptic training reduces suppression through contrast balancing to promote simultaneous use of both eyes, thus improving visual function (Li et al., 2013). Therefore, the research during this period provides an important foundation for motivating and guiding the subsequent binocular-based treatment.

Traditional amblyopia therapies are often monocular-based, including patching, atropine, and Bangerter filters (Papageorgiou et al., 2019). These therapies all force the use of the amblyopic eye by reducing visual quality of the non-amblyopic eye. However, these approaches face the challenges of inadequate patient adherence, the occurrence of reverse amblyopia, the limited efficacy, and the impact of life quality (Le and Orge, 2022; Maconachie and Gottlob, 2015; Wallace et al., 2013). These all compromise the treatment outcomes and reflect a necessity to promote the development of new treatment for amblyopia. In recent years, binocular digital therapy has become a new clinical avenue for amblyopia. It uses digital technologies such as tablet computers combined with red-green anaglyph glasses to present separate visual stimuli to each eye (Lamprogiannis et al., 2020; Tsani et al., 2024). The stimuli were modulated to balance interocular suppression and train binocular integration. The clinical recognition of binocular digital therapy has grown with accumulating evidence: the earlier version of Amblyopia PPP guidelines was cautious of binocular digital therapies due to limited data (Wallace et al., 2018), but the most recent version includes more affirmative statements based on recent trials (Cruz et al., 2023). Randomized controlled trials show that digital therapy is comparable to patching in improving amblyopia visual acuity after 2 and 16 weeks of treatment, but with higher adherence than patching (Jost et al., 2022; Wygnanski-Jaffe et al., 2023; Wygnanski-Jaffe et al., 2025). Additionally, other emerging therapies such as alternative flicker glass (Yuan et al., 2021) and non-invasive brain stimulation (Tuna et al., 2020) also offer new directions to treat amblyopia, but their efficacy requires more convincing evidence. For severe cases, combined therapies, such as a combination of traditional patching and novel binocular digital therapy, may be necessary to maximize therapeutic effect through multiple mechanisms. Our analysis of clinical trial trends (Figure 8A) further substantiates this therapeutic evolution, demonstrating that clinical research priorities have shifted to refining established monocular approaches and validating binocular digital therapies and other novel interventions. This reflects the field’s ongoing transition toward more effective and broadly applicable treatment strategies.

Although visual plasticity peaks during the critical period in early childhood, the emergence and exploration of various novel treatment methods for amblyopia have made the treatment population no longer limited to young children (Hensch and Quinlan, 2018; Thompson et al., 2024). A large part of high-impact (Table 5) and citation burst (Figure 7) literature now focuses on treating amblyopia in older children, teenagers, and adults. Analysis of the RCT populations (Figure 8B) also shows that clinical research efforts have expanded beyond the classical critical period. This expansion is driven by the development of diverse therapeutic approaches, including binocular digital therapy (Elhusseiny et al., 2021), combined patching and pharmacological treatment (Sharif et al., 2019), and novel neuromodulation techniques (El Salmawy et al., 2025). These facts indicate that treating amblyopia in older patients has become an important focus and a rapidly advancing area of research in this field.

## 5 Future perspectives

Based on the findings of this bibliometric analysis, several promising directions for future research in amblyopia treatment emerge. First, with the rapid development of technology, binocular digital therapies evolve across diverse platforms (e.g., iPad (Holmes et al., 2016) and Virtual Reality (Elhusseiny et al., 2021)) and engagement patterns [e.g., interactive gaming (Rajavi et al., 2019) and passive video viewing (Jost et al., 2022)]. Therefore, it is imperative for the future work to figure out the best use of these technologies for different populations that achieves the highest patient adherence and largest visual benefits with the lowest treatment burden.

Our analysis of RCTs shows that there is a notable shift toward incorporating a wider scope of outcome measures besides monocular and binocular visual functions (Figure 8C). However, there still lack the attention to the quality of life and functional vision of patients, which are also markedly impaired in amblyopia (Hatt et al., 2020). Therefore, these two aspects should be evaluated more comprehensively in the future. In addition, as shown in the figure, there is also a lack of research exploring the optimization of treatment regarding optimal dosing, duration, and personalized regimens. Rigorous studies are needed to establish evidence-based protocols tailored to different ages, amblyopia types, and severity to achieve truly personalized therapy.

Furthermore, the recent increase in exploration of neuromodulation techniques and other innovative approaches reflects a growing interest in expanding the therapeutic approaches. These interventions still need further rigorous clinical testing first to explore their efficacy, safety, and potential role as separate or adjunctive treatment.

## 6 Strengths and limitations

This study is the first to conduct a bibliometric analysis specifically focused on amblyopia treatment research, providing insights into knowledge structure and evolutionary trajectory of this field from 2015 to 2025. A supplementary analysis of RCTs from PubMed is also integrated to verify and clarify the hotspots and trends identified through the bibliometric data. This enhances the robustness and clinical relevance of our findings. The insights from this study offer valuable guidance for researchers in identifying promising directions and for clinicians in understanding the evolving evidence base for treatment options.

Despite its contributions, this study has some limitations. Firstly, the bibliometric analysis relies solely on publications indexed in the Web of Science core collection, which may exclude related publications in other databases. Secondly, we only included the research articles and reviews in English, potentially overlooking some research hotspots and introducing a geographic bias. Thirdly, due to the methodological approach itself, the citation analysis naturally favors earlier publications that have more time to accumulate citations, which may underrepresent the very recent but potentially high-impact literature. Finally, extending the analysis to a longer timeframe or comparing consecutive decades (e.g., 2005–2015 vs. 2015–2025) would offer a more complete picture of the evolutionary trajectory of amblyopia treatment.

## 7 Conclusion

This study conducted a bibliometric analysis of amblyopia treatment research from 2015 to 2025, supplemented by a review of RCTs. The current research hotspot is the development and clinical validation of binocular digital therapies, which represents a shift from traditional monocular treatment toward technology-driven and evidence-based binocular treatment. Future trends are expected to personalize treatment strategies by utilizing diverse technological platforms and incorporating more patient centered functional outcomes. Additionally, there is still ongoing exploration and clinical validation of other emerging therapies. This study provides valuable insights for directing future research and clinical management of amblyopia.

## Data Availability

The original contributions presented in this study are included in this article/supplementary material, further inquiries can be directed to the corresponding authors.
